# Optimization of Gearbox Fault Detection Method Based on Deep Residual Neural Network Algorithm

**DOI:** 10.3390/s23177573

**Published:** 2023-08-31

**Authors:** Zhaohua Wang, Yingxue Tao, Yanping Du, Shuihai Dou, Huijuan Bai

**Affiliations:** Department of Mechanical and Electrical Engineering, Beijing Institute of Graphic Communication, No. 1, Xinghua Street, Beijing 102600, China; wzh@bigc.edu.cn (Z.W.); taoyingxue111@126.com (Y.T.); doushuihai@bigc.edu.cn (S.D.); bhj07@bigc.edu.cn (H.B.)

**Keywords:** gearbox, fault detection, ResNeXt50 model, CBAM, time–frequency analysis method

## Abstract

Because of its long running time, complex working environment, and for other reasons, a gear is prone to failure, and early failure is difficult to detect by direct observation; therefore, fault diagnosis of gears is very necessary. Neural network algorithms have been widely used to realize gear fault diagnosis, but the structure of the neural network model is complicated, the training time is long and the model is not easy to converge. To solve the above problems and combine the advantages of the ResNeXt50 model in the extraction of image features, this paper proposes a gearbox fault detection method that integrates the convolutional block attention module (CBAM). Firstly, the CBAM is embedded in the ResNeXt50 network to enhance the extraction of image channels and spatial features. Secondly, the different time–frequency analysis method was compared and analyzed, and the method with the better effect was selected to convert the one-dimensional vibration signal in the open data set of the gearbox into a two-dimensional image, eliminating the influence of the redundant background noise, and took it as the input of the model for training. Finally, the accuracy and the average training time of the model were obtained by entering the test set into the model, and the results were compared with four other classical convolutional neural network models. The results show that the proposed method performs well both in fault identification accuracy and average training time under two working conditions, and it also provides some references for existing gear failure diagnosis research.

## 1. Introduction

The purpose of gearbox fault detection is to diagnose and prevent various abnormal states and fault states in a timely and correct manner, eliminate the impact of faults on equipment operation, improve the reliability, safety and effectiveness of equipment operation, and reduce the fault loss to the lowest level [1]. At present, gearbox fault detection can be roughly divided into three stages. The first stage is to rely on the experience and knowledge of professional and technical personnel to judge their working status; The second stage is fault detection technology with fault mechanism and signal processing as the core. However, the fault diagnosis methods in the first and second stages mostly rely on the expertise and experience of technicians. With the rapid development of pattern recognition and other disciplines, the third stage of intelligent fault detection with an artificial intelligence algorithm as the core has gradually developed, and the research into intelligent fault detection based on artificial intelligence algorithms has become increasingly in-depth.

At present, the third stage of gearbox intelligent fault detection methods with artificial intelligence as the core can be divided into two categories: the traditional machine learning-based method and the method based on deep learning [2,3]. The intelligent fault detection method based on traditional machine learning includes three processes: data acquisition, artificial feature extraction, and pattern recognition [4]. However, traditional machine learning methods generally do not have a deep architecture and are relatively closed themselves; therefore, the upper limit of the algorithm performance is low. Compared with traditional machine learning methods, the fault detection model based on deep learning is a typical end-to-end model with superior nonlinear mapping fitting ability, which can automatically extract data features in the optimization process. It makes up for the limitations of manual feature extraction in traditional machine learning methods and reduces the dependence on mechanism research and subjective experience [5,6,7]. To some extent, it improves the accuracy and intelligence of the diagnosis a–nd is more conducive to realization of the diagnosis of mechanical equipment faults on an automatic and large scale [8].

Today, in the field of troubleshooting, the most commonly used deep learning methods are deep belief networks (DBNs), stack auto-encoders (SAEs), recurrent neural networks (RNNs), generative adversarial networks (GANs) and convolutional neural networks (CNNs). Deep learning models are also being applied in the field of fault diagnosis. Xu et al. [9] used DBNs to extract the signal from the bearings and performed a rolling bearing fault detection. Shang et al. [10] proposed a rolling bearing fault detection model based on DBNs, and experiments proved that the model could accurately identify all kinds of faults and had good fault detection ability. Liu et al. [11] have proposed an effective deep learning approach called stacked auto-encoders (SAEs). This method extracted significant features directly from the frequency domain signal and eliminated the use of manual features to solve the problem of gearbox fault detection. Dai et al. [12] combined a sparse auto-encoder and a denoising auto-encoder, proposed the stacked sparse denoising auto-encoder diagnosis model, and applied the model to rolling bearing fault detection. Liu et al. [13] combined recurrent neural networks and autoencoders to realize the intelligent fault detection of rolling bearings. An et al. [14] used recurrent neural networks to realize the intelligent fault detection of bearings under time-varying working conditions. Wang et al. [15] used GAN in the fault diagnosis of rotating machinery in nuclear power plants to improve the generalization ability of the model when the data are inconsistent.

Compared with other deep learning models, the unique convolutional layer and pooled layer algorithm design of the CNN model can effectively reduce the complexity of the model, reduce the number of training parameters, and make the model easy to train and optimize. Common convolutional neural network models include the visual geometry group (VGG), GoogleNet, ResNet and ResNeXt. In order to improve the recognition accuracy of the model, the traditional convolutional neural network needs to deepen or widen the network, but with the deepening of the network layers, there will be gradient explosion or gradient disappearance, and network degradation will occur. As a variant of ResNet, the ResNeXt model adopts the idea of an inception network [16] and uses group convolution instead of traditional convolution to widen the network and reduce the number of training parameters of the model [17]. Because of its strong feature recognition ability, the ResNeXt model is widely used in the field of image recognition, such as: Yao Xiao et al. [18] proposed an automatic insect recognition system based on SE-ResNeXt, which realized the visual display of insect recognition results and the digital storage of insect data. Wang Guowei et al. [17] proposed an improved ResNeXt model to identify the occurrence degree of maize diseases. Pant Gaurav et al. [19] used an improved ResNeXt model to solve the problem of identification and classification of algae species. The above studies have proved the performance of the ResNeXt model in the field of image recognition, so this paper adopts the ResNeXt model as the gearbox fault identification and classification model.

However, when there is too much input, the ResNeXt model also becomes more complex and the training time is extended. In order to alleviate this problem, attention mechanisms are applied to neural network algorithms. Zheng et al. [20] proposed a multi-scale residual aggregation network by adding attention mechanisms to the network. Jiang et al. [21] applied the attention mechanism to predict the life of rolling bearings. Wu et al. [22] and Wu et al. [23] applied the attention mechanism to cross-domain fault detection, enabling neural network models to learn and update fault-related features in the training process, and improving the influence of the source domain with a stronger correlation. All the above studies show that the introduction of attention mechanisms into neural network models can effectively focus on significant information while ignoring redundant features, improving the feature extraction ability of neural network models for fault information, and thus reducing the amount of information processed by models and reducing computing resources.

In view of this, in this paper the ResNeXt model with strong feature extraction ability is selected as the basic model. The CBAM is embedded after the first convolutional layer and before the last convolutional layer of the model to highlight key information of different fault types and suppress useless information, and a CBAM-ResNeXt50 model is built. By comparing CWT and STFT, the effective method is selected to convert the vibration signal into time-frequency images as input to the CBM-ResNeXt50 model to reduce the influence of redundant background noise and improve the feature extraction ability of the model on the time-frequency images. The CBAM-ResNeXt50 model is compared with the original ResNeXt50 model and four other classical convolutional neural network models. The effectiveness of adding the CBAM and the robustness of the CBAM-ResNeXt50 model are verified by comparing the average training time and the accuracy of the test set of each model.

The organization structure was as follows:

- In Section 2, the theory of two time-frequency analysis methods, the basic principle of the CBAM, and the basic structure of the ResNeXt50 model are introduced.
- In Section 3, the CBAM-ResNeXt50 model integrated into the CBAM module is established and the flow of the algorithm is detailed.
- In Section 4, the proposed method is experimentally studied and verified using the open gearbox data set from Southeast University. The confusion matrix is obtained by comparing with four other classical convolutional neural networks. T-distributed stochastic neighbor embedding (t-SNE) is used to simplify the classification results into a two-dimensional plane and visualize them in the form of scatter plots. In addition, the anti-confusion capability of the proposed method is verified by the above methods.
- The conclusions of this study and future research directions are presented in Section 5.

## 2. Basic Theory

The main work of this paper is to add the CBAM block to the ResNeXt50 model to build the CBAM-RESNEXT50 model and to make the model pay more attention to the fault information. Vibration signals are converted into two-dimensional images by CWT and STFT, and the images are divided into a training set and a test set to train and test the constructed model. Compared to the original ResNeXt50 model and the four other classical convolutional neural network models, the performance of the proposed method is judged.

The second section is divided into three parts according to the main work, and respectively introduces the basic theories involved in the research, including the basic knowledge of CWT and STFT, the basic theory of attention mechanism, and the basic model structure of ResNeXt.

### 2.1. Time–Frequency Analysis Method

In the acquisition of vibration signals, there is a lot of noise interference, and it is difficult to train the network to judge the fault type directly according to the obtained signals. It is necessary to use the method of time–frequency analysis and processing to make the characteristics of the fault prominent. Time–frequency analysis methods include short-time Fourier transform (STFT) and continuous wavelet transform (CWT) [24].

In 1946, Dennis Gaor proposed the STFT algorithm [25]. The basic idea is: In the framework of the traditional Fourier transform, the nonstationary signal is regarded as the superposition of a series of short-time stationary signals. The short-time is realized by adding windows in the time domain, after which it is transformed into a local spectrum in a very small period time near the time t. The whole time domain is covered by translation parameters, that is, the local spectrum of any position is obtained by moving in the whole time domain with the change in time t, and then the time–frequency–energy analysis is carried out [26].

For a given non-stationary signal, the STFT of the signal $s(t)\in L^{2}(R)$ is defined as:

(1) $${STFT}_{S}(t,\omega )=\int_{-\infty }^{+\infty }s(\tau )h(\tau -t)e^{-i\omega t}d\tau$$

where: h(t) is the window function.

It can be seen from Equation (1) that, in the STFT, the translation parameter t is first used to segment signal s(t) through window function h(t), namely:

(2) $$s_{t}=s(\tau )h(\tau -t)$$

The signal at time τ is obtained near time *t*, and the Fourier transform is then applied to that signal $s_{t}(\tau )$.

The time resolution and frequency resolution of STFT are contradictory. The higher the time resolution is, the lower is the frequency resolution, and vice versa, which causes STFT to have certain limitations when analyzing high frequency signals. In addition, the analysis results of STFT are affected by the window function. Different window functions will produce different analysis results, so it is necessary to choose the appropriate window function according to the specific situation.

CWT [27] is derived from the decomposition and reconstruction of the input signal using a finite length or fast attenuation ”mother wavelet”, and the mother wavelet fits the input signal through scale contraction and wavelet translation. The original signal in the time–domain y(x) is decomposed into a series of wavelet transform coefficients λ(k,x) after inner product operation by CWT through a specific mother wavelet φ(x), so as to construct a localized time–frequency signal with good time–domain and frequency domain.

For any square integrable function $y(x)\in L^{2}(R)$, its CWT is defined as [28]:

(3) $$\lambda (k,x)=\int y(x)\varphi_{k,\tau }^{*}(x)dx$$

where, $\varphi_{k,\tau }^{*}(x)$ is the conjugate operation of the wavelet basis function $\varphi_{k,\tau }(x)$.

A series of wavelet basis functions can be obtained by stretching and time-delaying the transformation of the mother wavelet φ(x). If we allow $\varphi (x)\in L^{2}(R)$, its process can be expressed as:

(4) $$\varphi_{k,\tau }=\frac{1}{\sqrt{k}}\varphi (\frac{x-\tau }{k}),\;k,\tau \in R,k>0$$

where *k* is the scale parameter, when *k* > 1, the stretching of φ(x) is realized, and when 0 < *k* < 1, the compression of φ(x) is realized. τ is the translation parameter, which specifies the position of the translation of the wavelet function along the time axis. *k* cannot be negative, so the normalization unified treatment becomes positive, and ${\left|k\right|}^{-1/2}$ realizes the normalization processing of the signal capability.

Compared with the STFT, CWT has a different size time–frequency window at different times and frequencies, which can achieve higher frequency resolution in the low frequency region. However, the time–frequency window of CWT is not completely adaptive; it also needs to choose a suitable mother wavelet.

### 2.2. Attention Mechanism

**Figure 1:**
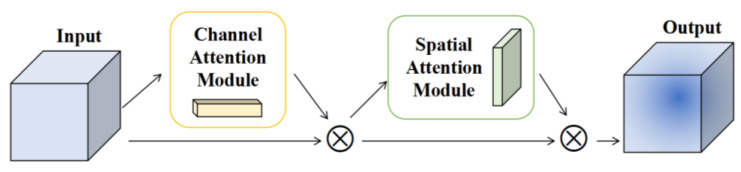
The structure of CBAM.

**Figure 2:**
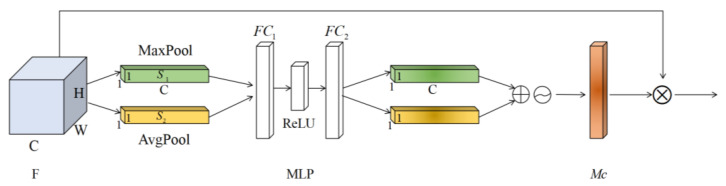
Channel Attention Module.

An attention mechanism is a way to realize adaptive attention in neural networks. Generally speaking, it makes the network pay more attention to the effective unit and suppresses the invalid unit in the feature extraction process [29]. CBAM [30] is a lightweight attention module, which is composed of a channel attention module and a spatial attention module. It can perform the attention mechanism along the channel and space, infer the weight coefficient, and then multiply the feature map [31]. Its structure is shown in Figure 1:

Channel attention module. Channel attention is the feature on which channel it is meaningful to focus, as shown in Figure 2.

Suppose that the input feature map (*C*, *W*, *H*) is denoted as *F*, where *W* is the width of the input feature map, *H* is the height of the input feature map, and *C* is the number of channels in the input feature map. The global spatial information of *F* is compressed through the maximum pooling layer and the average pooling layer to generate two feature maps $s_{1}$ and $s_{2}$, both of which are 1 × 1 × *C*. $s_{1}$ and $s_{2}$ obtained two one-dimensional feature maps by sharing a multi-layer perception (MLP) consisting of the fully connected layer $FC_{1}$, $FC_{2}$ and ReLU functions. After summing the two one-dimensional feature graphs according to channels, the Sigmoid function is used to normalize them, and the weighted statistic value Mc of each channel size 1 × 1 × *C* is obtained. The process can be expressed by Formula (5):

(5) $$\begin{array}{l} Mc\left(F\right)=\sigma \left\{MLP\left[\left.Avgpool\left(F\right)\right]+MLP\left[Maxpool\left(F\right)\right]\right.\right\}\\ \;=\left\{MLP\left[\frac{1}{H\times W}\sum_{x_{0}=1}^{H}\sum_{y_{0}=1}^{W}f_{k_{0}}(x_{0},y_{0})\right]\right\}+MLP\left[\max f_{k_{0}}(x_{0,}y_{0})\right] \end{array}$$

where, σ represents the Sigmoid function, and $f_{k_{0}}(x_{0},y_{0})$ represents the pixel value of the point whose coordinate is $x_{0}{,y}_{0}$ in the $k_{0}$ channel of the input feature graph *F*.

**Figure 3:**
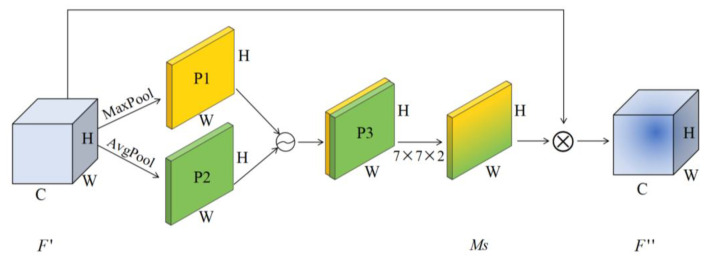
Spatial Attention Module.

Spatial attention mechanism. After the output of channel attention, the spatial attention module is introduced to focus on which part of the space features are meaningful, as shown in Figure 3.

The spatial attention mechanism takes the feature map $F^{\prime \prime }$ output by the channel attention module as the input of this module and carries out global maximum pooling and global average pooling, respectively, on the channel dimension to obtain two channel descriptions $P_{1}$ and $P_{2}$. The two descriptions are concatenated together according to the channels to obtain the feature description $P_{3}$. The convolutional layer is used to encode and fuse the information of different positions in $P_{3}$, and the spatial weighted information *Ms* is obtained. Finally, we multiply the weight coefficient and feature $F^{\prime }$ to obtain the new feature after scaling. The process can be expressed by Formula (6):

(6) $$Ms(F)=\sigma \left\{f^{7\times 7}\left[Avgpool(F^{\prime });Maxpool(F^{\prime })\right]\right\}$$

where: $f^{7\times 7}$ represents a convolution layer with a convolution kernel of size 7 × 7.

CBAM attention mechanism [32]. Multiply the input feature map *F* with the corresponding elements of the two matrices of the weight value *Mc* of each channel, carry out the feature recalibration of *F*, and obtain the feature mapping $F^{\prime }$ which can effectively reflect the feature key channel information. On the basis of channel weighting, the series spatial attention mechanism is used to carry out adaptive weighting on the spatial feature information. $F^{\prime }$ is taken as the input to the spatial attention module and multiplied by the corresponding elements of the two matrices of the spatial weight coefficient *Ms* to obtain the significant feature graph $F^{\prime }$ containing the channel location information and spatial location information. The network can pay more attention to the strong input features of the gearbox two-dimensional image, and improve its spatial feature selection ability. Its process can be expressed by the formula:

(7) $$\begin{array}{c} F^{\prime }=Mc⊗F\\ F^{\prime \prime }=Ms⊗F^{\prime } \end{array}$$

### 2.3. ResNeXt Model

Residual Block. By calculating residuals through identity mapping, part of the upper-layer information can be directly transmitted to the last layer, which is conducive to the back propagation of the gradient.

If we suppose that the input sample of part of the neural network is *x*, F(x) represents the residual error, and the two together form the expected output H(x). As shown in Formula (8), H(x)=x forms the identity mapping when F(x)=0.

(8) H(x)=F(x)+x→F(x)=H(x)−x

The Bottleneck Structure. The bottleneck has two structures. The two structures correspond to two situations: Different input and output channel counts (Bottleneck1), and identical input and output channel counts (Bottleneck2).

Bottleneck1 has four mutable parameters *C*, *W*, *C*_1_, and *S*. Compared with Bottleneck2, Bottleneck1 has one more right-side convolution layer, which is designated as a function H(x). Bottleneck1 corresponds to the different input *x* and output F(x) channel numbers, and it is the added convolutional layer that changes *x* into H(x), which matches the input and output dimensional differences (H(x) and F(x) have the same number of channels).

Bottleneck2 has two mutable parameters *C* and *W*. Assume that the shape of the input *x* is (*C*, *W*, *W*), and the three convolution blocks (along with the related BN and ReLU) to the left of Bottleneck2 are the function F(x). After adding the two together (F(x)+x) and passing through a ReLU function, the output of Bottleneck2 is obtained. The shape of the output remains (*C*, *W*, *W*), namely, Bottleneck2 corresponds to situations where the number of input and output channels is identical.

ResNeXt Block. The ResNeXt network architecture uses the stack of VGG networks and the idea of partition and integration inception, using a block with the same structure for the stack [33]. The essence of ResNeXt is grouping convolution, controlling the number of groups through cardinality [34]. The ResNeXt block divides the input channels into groups, then each group performs a convolution calculation with its own convolution kernel, and finally merges the output of all groups by a 1 × 1 convolution. Because the ResNeXt block has the same topology, it can be used directly to replace the remaining blocks in the regular residual network and stack them to construct the desired structure.

## 3. Gearbox Fault Detection Method Based on the CBAM-ResNeXt50 Model

**Figure 4:**
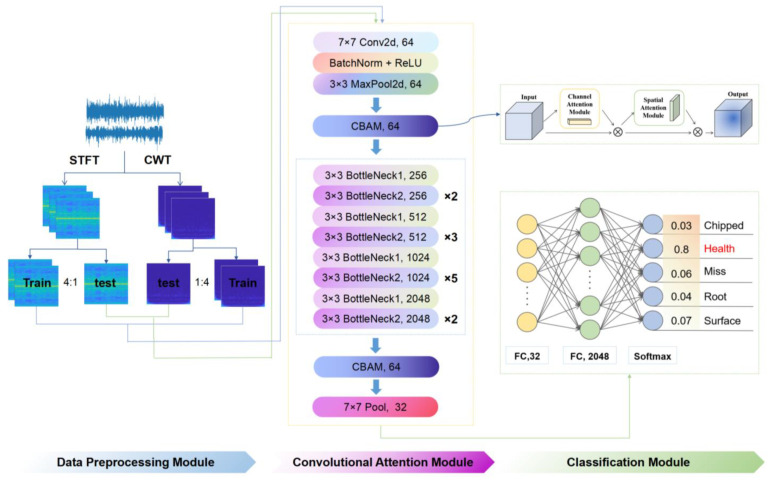
Algorithm flow chart.

The data set in this paper was a labeled data set with four fault categories and one normal category, each of which was distinguished according to the different characteristics of the fault. The flow of fault diagnosis method based on the CBM-ResNeXt50 model is shown in Figure 4. Firstly, one-dimensional vibration signals of two working conditions in the data set were converted into different two-dimensional images as sample data by CWT and STFT, and the sample data were divided into the training sets and test sets according to 4:1 by labeling. The training set was used as input to the CBAM-ResNeXt50 model and features were extracted. The extracted features were then input into the CBAM to make the model pay more attention to the faulty areas in the time–frequency graph. In addition, the CBAM information output was input into the global average pooling layer and the full connection layer. Furthermore, the fault categories of time–frequency images were output and the trained CBAM-ResNeXt50 model was saved. In the classification module shown in Figure 4, the output result is the probability of five failure types, including “Chipped” 0.03, “Health” 0.8, “Miss” 0.06, “Root” 0.04, and “Surface” 0.07. Because the probability of Health is the highest, this gear is judged to be “Health”. Finally, the test sets under the two conditions were input into the trained network model, respectively, for testing to obtain the accuracy of the test set. The classification scatter diagram obtained by the t-SNE technique and the confusion matrix diagram were used to judge the merits and demerits of this model.

## 4. Experimental Research

### 4.1. Experimental Data and Setting of Parameters

**Figure 5:**
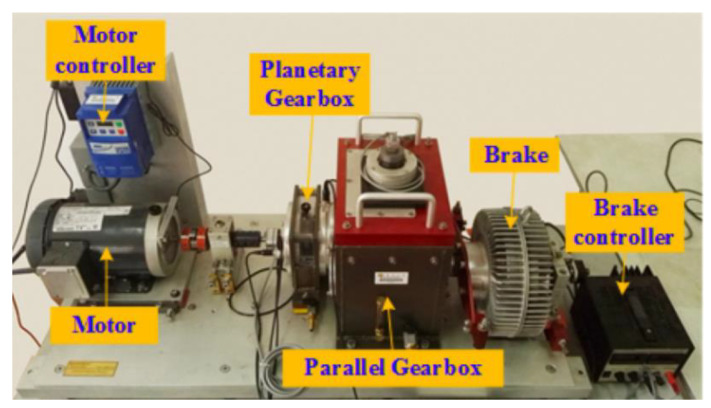
Experimental setup for gearbox.

The experimental data are obtained from the drivetrain dynamic simulator (DDS) of Southeast University [35]. The platform is shown in Figure 5 and mainly consists of six sections: motor, motor controller, planetary gearbox, parallel gearbox, brake, and brake controller. The whole experiment was carried out under two working conditions according to different settings of speed and load. The speed was set at 20 Hz and 30 Hz, and the load was set at 0 V and 2 V while the signals of four kinds of gear fault state and the signal of one kind of gear health state were collected. Each state signal includes the vibration signal of the motor, motor torque, planetary gearbox in *x*, *y*, and *z* directions, and parallel gearbox in *x*, *y*, and *z* directions. The highest frequency of the collected signal was 2000, and the sampling frequency was 5120 Hz, namely, 5120 sampling points within 1 s. Four types of gear failure data include chipped, miss, root, and surface. The detailed data set type division is shown in Table 1.

**Figure 6:**
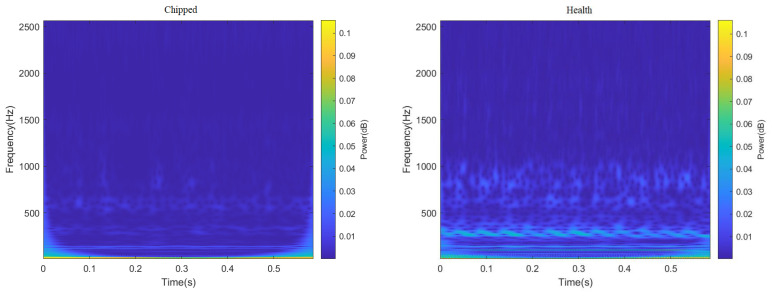

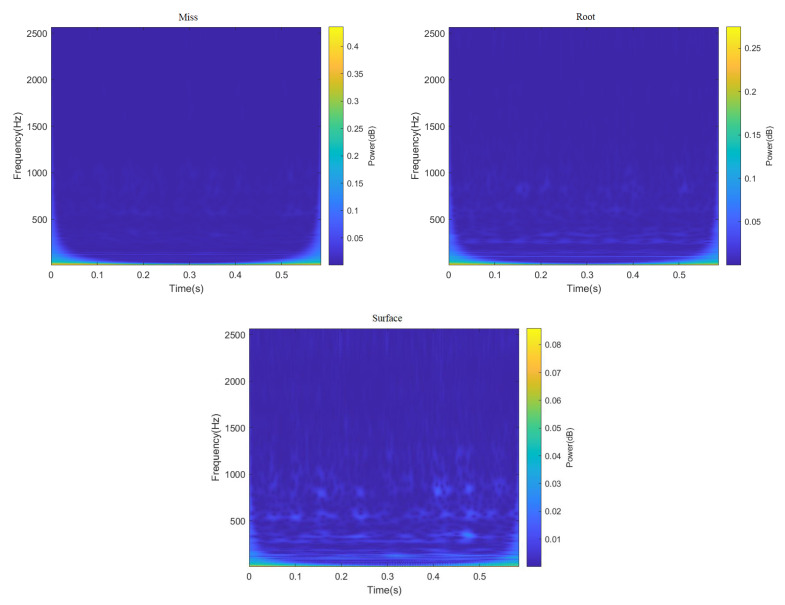
Time–frequency decomposition diagram of gear vibration signals with different fault types after passing through CWT.

**Figure 7:**
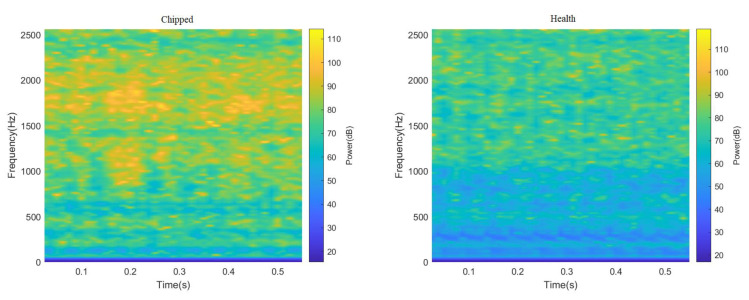

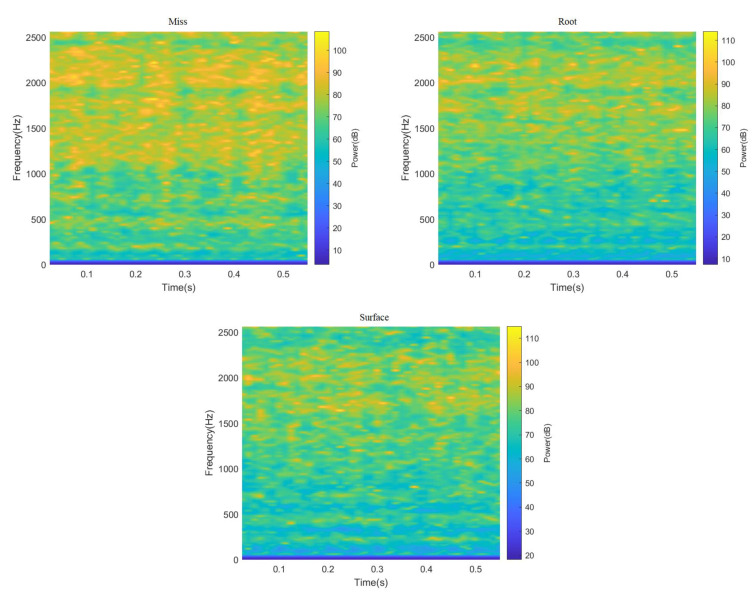
Spectrogram of gear vibration signals with different fault types after passing through STFT.

ResNeXt model is widely used in the field of image recognition due to its strong feature recognition ability. Therefore, this paper truncated all 3000 data points in the data set, and sample graphs with a size of 224 × 224 RGB three-channel through STFT and CWT were generated, respectively, to enhance the feature extraction capability of the ResNeXt model for the image. Finally, the sum of 4000 two-dimensional images of different fault categories under two working conditions was obtained, part of which is shown in Figure 6 and Figure 7. It can be seen from Figure 6 and Figure 7, after STFT and CWT transformation, that the signal used was stable, and that images of various fault types have different characteristics.

In STFT transformation, the select window type was the Hanning window with size 256 and moving step size 64. Because a complex morlet wavele adopted a Gaussian function with minimum time–frequency window area and had good localization performance in the time–frequency domain and good symmetry, a complex morlet wavele was selected as the parent wavelet in this paper. The mathematical expression of the complex morlet wavele is:

(9) $$\varphi (t)=\frac{1}{\sqrt{\pi f_{b}}}e^{2i\pi f_{c}t}e^{\left(\frac{{-t}^{2}}{f_{b}}\right)}$$

where $f_{b}$ is the bandwidth of the wavelet, $f_{c}$ is the center frequency of the wavelet $and\;2\pi f_{c}\geq 5$.

All experiments in this paper were performed on the same platform. The experimental operating environment was ubuntu20.04 OS, the GPU was RTX 3080(10 GB), and the CPU was 12-core Intel(R) Xeon(R) Platinum 8255C. During the experiment, the parameters were adjusted and used as shown in Table 2. The network training epoch was set to 50. In the training process, the initial value of the learning rate was set to 0.001, and the automatic adjustment strategy was adopted to realize the automatic adjustment of the learning rate. The dropout value was set to 0.2. All models were trained using adaptive moment estimation (Adam).

### 4.2. Comparison of Time–Frequency Analysis Method

**Figure 8:**
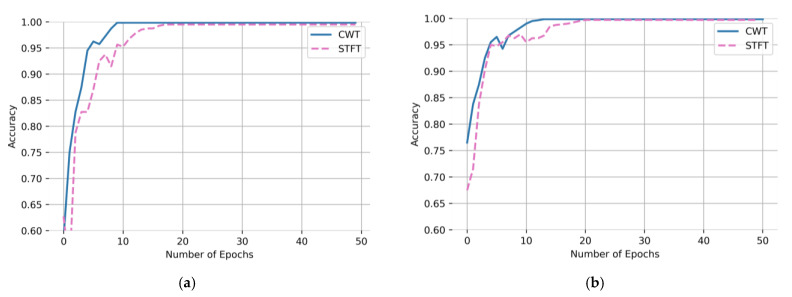
Comparison of CWT and STFT under two working conditions (**a**) Working Condition 1: 20 Hz–0 V, (**b**) Working Condition 2: 30 Hz–2 V.

In this paper, two-dimensional images of CWT and STFT would be used as the input of the CBAM-ResNeXt50 model, respectively. Figure 8 is a comparison of the accuracy and convergence rate of the model test set obtained. As can be seen from Figure 8, the accuracy and convergence speed of the test set after CWT were better than the STFT, which is more suitable for model training. Therefore, this article used it as a time–frequency analysis method to carry out the time–frequency transformation of one-dimensional time series fault signals of the gearbox.

### 4.3. Model Training and Result Analysis

**Figure 9:**
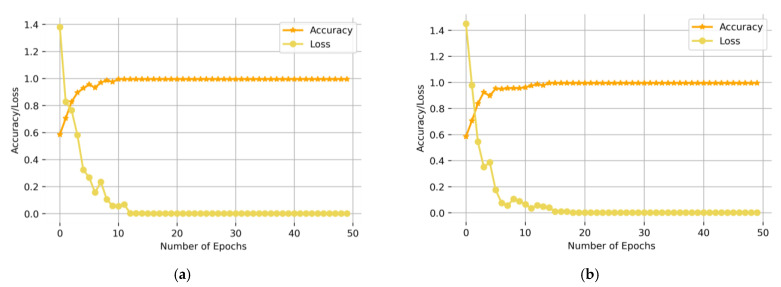
Accuracy and loss rate convergence curves of CBAM-ResNeXt50 under working conditions (**a**) Working condition 1: 20 Hz–0 V, (**b**) Working condition 2: 30 Hz–2 V.

After constructing the network, the classification prediction results were obtained through 50 training epochs. The training convergence curves of the CBAM-ResNeXt50 model under two working conditions are shown in Figure 9. As can be seen from Figure 9, the error in the network decreased gradually with the increase in the epoch. After 10 epochs, the network converged completely. At this time, the network accuracy rate reached 99.95% and 99.875%, and the loss reached 0.0015 and 0.0135, respectively. It can be seen from the above that the CBAM-ResNeXt50 model had a good performance in fault classification.

**Figure 10:**
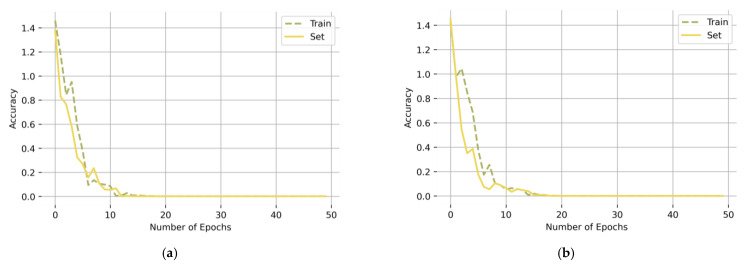
Accuracy of the training and test sets under working conditions (**a**) Working Condition 1: 20 Hz–0 V, (**b**) Working condition 2: 30 Hz–2 V.

For complex tasks with a limited sample size but requiring the use of a strong model, the model is prone to overfitting phenomena with a small loss on the training set and a large loss on the test set. Figure 10 shows the loss rates of the training set and the test set of the CBAM model under two working conditions. As can be seen from Figure 10, the loss rates of the model test set under the two working conditions are very small, and there is no overfitting problem.

To demonstrate the effectiveness of CBAM in the fusion model in this paper, comparative experiments were conducted on whether to use the CBATM module. Among them, CBAM-ResNeXt50 represented the ResNeXt50 model integrated into CBAM, and ResNeXt50 represented the original model not integrated into CBAM. At the same time, the performance of classical convolutional neural networks such as the DenseNet121, the ResNet50, and the AlexNet was compared.

Table 3 shows the results of the training time and fault recognition accuracy of each model. It can be seen from Table 3 that the accuracy of the CBAM-ResNeXt50 model reached 99.95% and 99.875%, respectively, under two working conditions. The addition of CBAM significantly improved the accuracy of the ResNeXt50 network in identifying different faults. The experiment proved the effectiveness of CBAM for gearbox fault identification.

**Figure 11:**
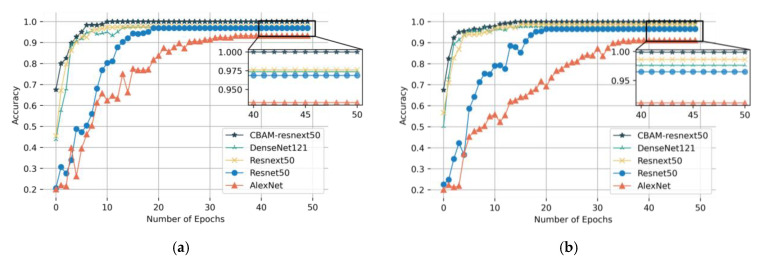
Training convergence curves of five models under working conditions (**a**) Working Condition 1: 20 Hz–0 V, (**b**) Working condition 2: 30 Hz–2 V.

Figure 11a,b shows the accuracy curves of five model test sets. Under the two working conditions, with an increasing number of epochs, the increasing trend of test accuracy of different models was different. After about 10 epochs, the CBAM-ResNeXt50 model began to converge, the DenseNet121 and ResNeXt50 models began to converge after small oscillations, while the ResNet50 and AlexNet models still did not converge. Comparing several models, we see that CBAM could improve the diagnostic accuracy and convergence speed of the model.

**Figure 12:**
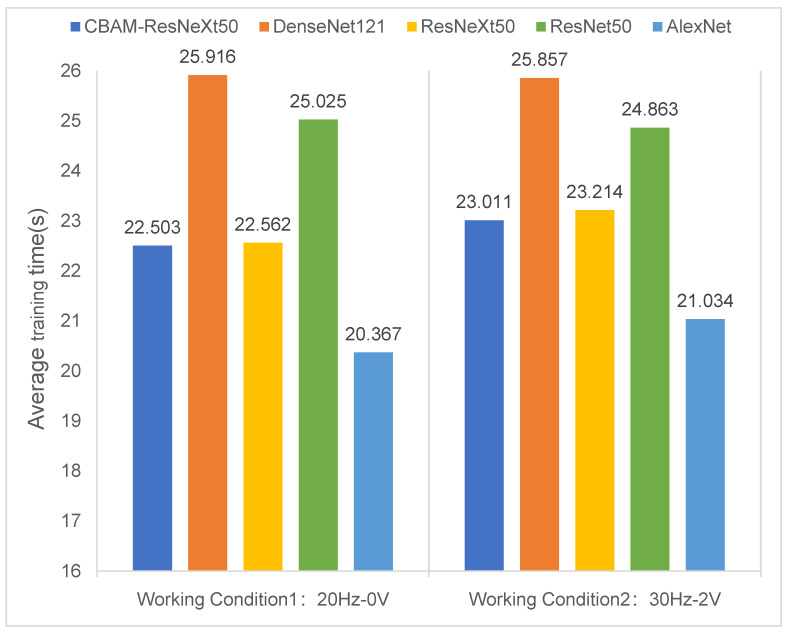
Comparison of the average training time of five models under two working conditions.

Figure 12 compared the average training time of five neural network models under two working conditions. The number of training epochs was 50, and the sample sizes of the training and test data sets were 3200 and 800. As can be seen in Figure 12, the average running time of the CBAM-ResNeXt50 model was shorter than those of the other three models due to the integration of CBAM. This indicated that the addition of CBAM can shorten the time of the model training on the premise of improving the accuracy of the model training. However, the AlexNet model had a single structure and only used a few convolution layer parameters, so the shortest average training time was reasonable. Although the AlexNet model took the shortest time, its model training accuracy was low and could not converge quickly.

**Figure 13:**
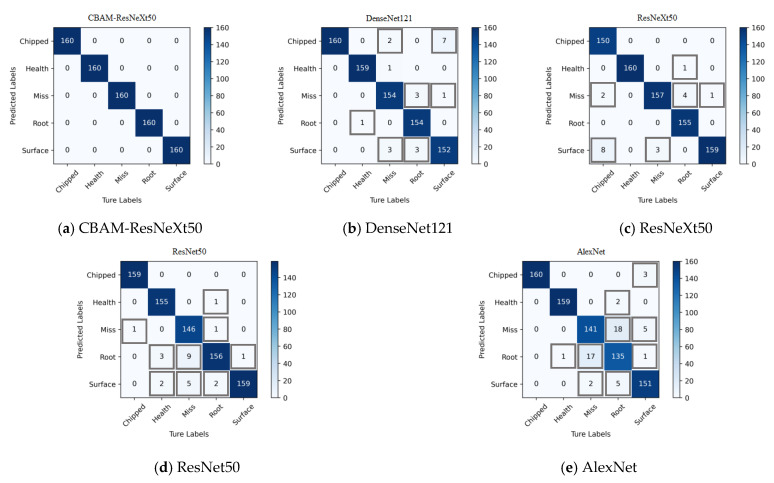
Confusion matrix for fault detection of five models under working condition 1.

**Figure 14:**
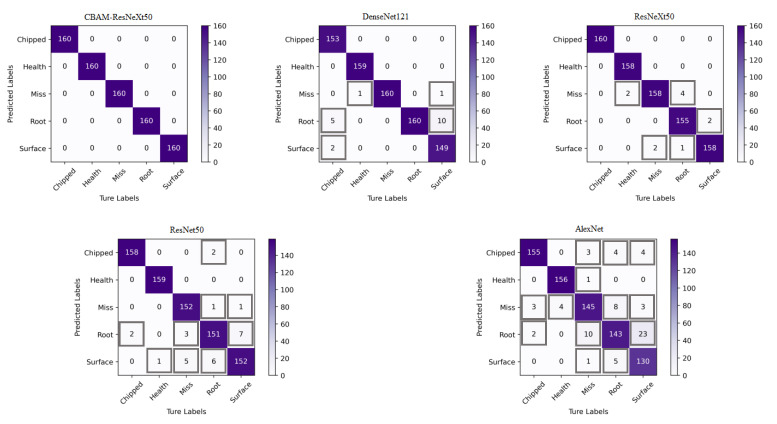
Confusion matrix for fault detection of five models under working condition 2.

The two models were verified by using the two-dimensional images obtained by CWT. Figure 13 and Figure 14 show the confusion matrix obtained by fault classification by the CBAM-ResNeXt50 model and the ResNeXt50 model under two working conditions, respectively. The abscissa represented the real fault and the ordinate represented the predicted result, which was, respectively, chipped, health, miss, root, and surface. The depth of the color represents the number of identified samples. The darker the color is, the greater is the number of correctly identified samples, and the greater is the accuracy.

In this paper, the average precision (AP) and average recall (AR) of five fault types were used to analyze the confusion matrix obtained. The precision represented the ratio of correct samples to the total samples in the prediction results. The recall rate represented the ratio of the number of predicted samples to the total number of actual samples [36].

The gearbox data set published by Southeast University was used to conduct experiments on the CBAM-ResNeXt50 model, the ResNeXt50 model, and three other convolutional network models. As can be seen from Table 4, the DenseNet121 and ResNeXt50 model both had an average precision and recall rate of around 97%, the average precision and recall rate of the ResNet50 model were both around 96%, and the average precision and recall rate of the AlexNet model were both about 93%. Based on the original ResNeXt50 model, CBAM was integrated into the method proposed in this paper, which optimized the average precision rate and the average recall rate of gear fault type detection in the model.

**Figure 15:**
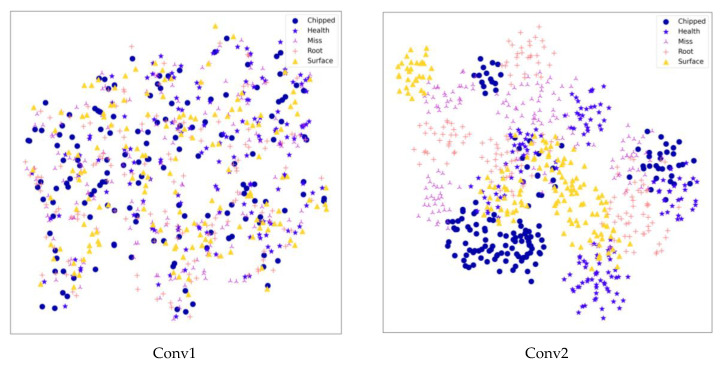

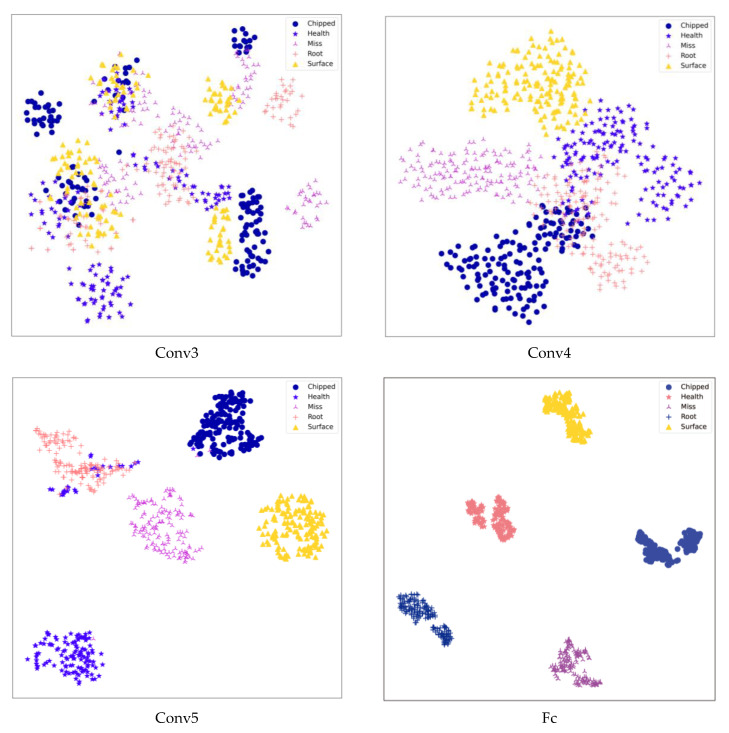
Dimension reduction results of each layer of the CBAM-ResNeXt50 model.

Using the t-SNE algorithm, the prediction of the CBAM-ResNeXt50 model on the test set could be displayed visually, so that the multidimensional output prediction data could be displayed in the two-dimensional space. t-SNE technology was used to reduce the feature dimension of trained data in data set 1. The result is shown in Figure 15. Dimensionality reduction visualization showed that at the beginning of classification, different fault data were mixed and difficult to distinguish. After training, the division between classes gradually became clear and there were five clear distributions in the entire connection layer.

## 5. Conclusions

This paper presented a fault detection method for the gearbox based on the CBAM-ResNeXt50 model. Compared with other existing convolutional neural network models, a CBAM attention mechanism was added to the ResNeXt basic model in this paper, which made the model pay more attention to fault information during training. We used the gearbox data set published by Southeast University to verify the model proposed in this paper. The results show that under the two working conditions, the accuracy of the test set was 100% and 99.875%, and the average training time was 22.619 s and 23.083 s. We comprehensively compared this result with four models, including DenseNet121, ResNeXt50, ResNet50, and AlexNet, and found that the performance of the proposed method in this paper was more stable. The model parameters and experimental results provided in this paper also provide some data reference for existing fault diagnosis research. However, the existing gear fault data are few, difficult to collect, and the model training requires a large amount of data, so in the case of a small amount of data, using neural network model training and achieving good stability is one of the future research directions.

## Figures and Tables

**Table 1:** Data set division of gearbox of DDS experimental platform.

	Type	Health	Chipped	Miss	Root	Surface	Total	Working Conditions
Dataset 1	Training	640	640	640	640	640	4000	20 Hz–0 V
	Validation	160	160	160	160	160		
Dataset 2	Training	640	640	640	640	640	4000	30 Hz–2 V
	Validation	160	160	160	160	160		

**Table 2:** Parameter selection.

Hyper Parameter	Values
Epoch	50
Batch_size	32
Learning rate	0.001
Learning rate decay	0.1
Dropout rate	0.2
Adam	-

**Table 3:** Training results of five models under two working conditions.

Working Conditions	Neural Network Model	Accuracy (%)
Condition 1 20 Hz–0 V	CBAM-ResNeXt50	99.95%
	ResNeXt50	97.625%
	ResNet50	96.875%
	DenseNet121	97.25%
	AlexNet	93.25%
Condition 2 30 Hz–2 V	CBAM-ResNeXt50	99.875%
	ResNeXt50	98.75%
	ResNet50	96.5%
	DenseNet121	97.625%
	AlexNet	91.125%

**Table 4:** Evaluation indexes of confusion matrices of five models under working condition 1.

Neural Network Model	Average Precision		Average Recall	
	Working Condition 1	Working Condition 2	Working Condition 1	Working Condition 2
CBAM-ResNeXt50	1.0	1.0	1.0	1.0
DenseNet121	0.9741	0.9778	0.9738	0.9776
ResNeXt50	0.9747	0.9864	0.9738	0.9863
ResNet50	0.9699	0.9655	0.9687	0.9650
AlexNet	0.9322	0.9152	0.9325	0.9113

## Data Availability

The public datasets used in this experiment can be obtained at: https://gitee.com/zhengkun110/Mechanical-datasets/tree/master/gearbox/gearset (accessed on 15 March 2023).

## References

[1] Li C., Zhang S., Qin Y., Estupinan E. (2020). A systematic review of deep transfer learning for machinery fault detection. Neurocomputing.

[2] Jiao J., Zhao M., Lin J., Liang K. (2020). A comprehensive review on convolutional neural network in machine fault detection. Neurocomputing.

[3] Lei Y., Lin J., Zuo M., He Z. (2014). Condition monitoring and fault detection of planetary gearboxes: A review. Measurement.

[4] Sun T., Yu G., Gao M., Zhao L., Bai C., Yang W. (2021). Fault detection Methods Based on Machine Learning and its Applications for Wind Turbines: A Review. IEEE Access.

[5] Yang J., Guo Y., Zhao W. (2019). Long short-term memory neural network based fault detection and isolation for electro-mechanical actuators. Neurocomputing.

[6] Ding Y., Ma L., Ma J., Suo M., Tao L., Cheng Y., Lu C. (2019). Intelligent fault detection for rotating machinery using deep Q-network based health state classification: A deep reinforcement learning approach. Adv. Eng. Inform..

[7] Wang Y., Wu D., Yuan X. (2020). LDA-based deep transfer learning for fault detection in industrial chemical processes. Comput. Chem. Eng..

[8] Chen Z., Mauricio A., Li W., Gryllias K. (2020). A deep learning method for bearing fault detection based on Cyclic Spectral Coherence and Convolutional Neural Networks. Mech. Syst. Signal Process.

[9] Xu F., Fang Y., Wang D., Liang J., Tsui K. (2018). Combining DBN and FCM for fault detection of Roller Element Bearings without Using Data Labels. Shock Vib..

[10] Shang Z., Liao X., Geng R., Gao M., Liu X. (2018). Fault detection method of rolling bearing based on deep belief network. J. Mech. Sci. Technol..

[11] Liu G., Bao H., Han B. (2018). A Stacked Autoencoder-Based Deep Neural Network for Achieving Gearbox fault detection. Math. Probl. Eng..

[12] Dai J., Tang J., Shao F., Huang S., Wang Y. (2019). Fault detection of Rolling Bearing Based on Multiscale Intrinsic Mode Function Permutation Entropy and a Stacked Sparse Denoising Autoencoder. Appl. Sci..

[13] Liu H., Zhou J., Zheng Y., Jiang W., Zhang Y. (2018). Fault detection of rolling bearings with recurrent neural network-based autoencoders. ISA Trans.

[14] An Z., Li S., Wang J., Jiang X. (2020). A novel bearing intelligent fault detection framework under time-varying working conditions using recurrent neural network. ISA Trans.

[15] Wang Z.C., Xia H., Yin W.Z., Yang B. (2023). An improved generative adversarial network for fault diagnosis of rotating machine in nuclear power plant. Ann. Nucl. Energy.

[16] Liu Y., Dou S., Du Y., Wang Z. (2023). Gearbox fault detection Based on Gramian Angular Field and CSKD-ResNeXt. Electronics.

[17] Wang G., Wang J., Yu H., Sui Y. (2022). Research on Identification of Corn Disease Occurrence Degree Based on Improved ResNeXt Network. Int. J. Pattern Recognit. Artif. Intell..

[18] Xiao Y., Zhou A., Zhou L., Zhao Y. (2023). Automatic insect identification system based on SE-ResNeXt. Int. J. Syst. Control Commun..

[19] Pant G., Yadav D.P., Gaur A. (2020). ResNeXt convolution neural network topology-based deep learning model for identification and classification of Pediastrum. Algal Res..

[20] Zheng Q., Chen Z., Liu H., Lu Y., Li J., Liu T. (2023). MSRANet: Learning discriminative embeddings for speaker verification via channel and spatial attention mechanism in alterable scenarios. Expert Syst. Appl..

[21] Jiang C., Liu X., Liu Y., Xie M., Liang C., Wang Q. (2022). A Method for Predicting the Remaining Life of Rolling Bearings Based on Multi-Scale Feature Extraction and Attention Mechanism. Electronics.

[22] Wu Z., Jiang H., Zhu H., Wang X. (2023). A knowledge dynamic matching unit-guided multi-source domain adaptation network with attention mechanism for rolling bearing fault detection. Mech. Syst. Signal Process..

[23] Wu H., Li J., Zhang Q., Tao J., Meng Z. (2022). Intelligent fault detection of rolling bearings under varying operating conditions based on domain-adversarial neural network and attention mechanism. ISA Trans..

[24] Zhang Q., Deng L. (2023). An Intelligent fault detection Method of Rolling Bearings Based on Short-Time Fourier Transform and Convolutional Neural Network. J. Fail. Anal. Prev..

[25] Liu Q., Xu S.Q., Cao X.Y. (2022). Research on residual life prediction of rolling bearings based on STFT-CNN. Testing Const..

[26] Zhou S., Xiao M., Bartos P., Filip M., Geng G. (2020). Remaining Useful Life Prediction and fault detection of Rolling Bearings Based on Short-Time Fourier Transform and Convolutional Neural Network. Shock Vib..

[27] Aguiar-Conraria L., Soares M. (2011). The Continuous Wavelet Transform: A Primer.

[28] Chen S.L., Li J.L. (2012). The contract finite element analysis for the small module gear of the car door lock devices. J. Mech. Transm..

[29] Li Z., Li B., Ni H., Ren F., Lv S., Kang X. (2022). An Effective Surface Defect Classification Method Based on RepVGG with CBAM Attention Mechanism (RepVGG-CBAM) for Aluminum Profiles. Metals.

[30] Woo S., Park J., Lee J.Y., Kweon I.S. CBAM: Convolutional block attention module. Proceedings of the European Conference on Computer Vision.

[31] Cui W., Meng G., Gou T., Wang A., Xiao R., Zhang X. (2022). Intelligent Rolling Bearing fault detection Method Using Symmetrized Dot Pattern Images and CBAM-DRN. Sensors.

[32] Chen C., Wu B., Zhang H. (2023). An Image Recognition Technology Based on Deformable and CBAM Convolution Resnet50. IAENG Int. J. Comput. Sci..

[33] Xie S., Girshick R., Dollár P., Tu Z., He K. Aggregated Residual Transformations for Deep Neural Networks. Proceedings of the IEEE Conference on Computer Vision and Pattern Recognition (CVPR).

[34] Liang Z., Tao M., Wang L., Su J., Yang X. (2021). Automatic Modulation Recognition Based on Adaptive Attention Mechanism and ResNeXt WSL Model. IEEE Commun. Lett..

[35] Shao S., Stephen M., Yan R., Pierre B. (2018). Highly accurate machine fault detection using deep transfer learning. IEEE Trans. Ind. Informatics.

[36] Fränti P., Mariescu-Istodor R. (2023). Soft precision and recall. Pattern Recognit. Lett..

